# The L-type Ca^2+^ Channels Blocker Nifedipine Represses Mesodermal Fate Determination in Murine Embryonic Stem Cells

**DOI:** 10.1371/journal.pone.0053407

**Published:** 2013-01-08

**Authors:** Filomain Nguemo, Bernd K. Fleischmann, Manoj K. Gupta, Tomo Šarić, Daniela Malan, Huamin Liang, Kurt Pfannkuche, Wilhelm Bloch, Heribert Schunkert, Jürgen Hescheler, Michael Reppel

**Affiliations:** 1 Institute of Neurophysiology, University of Cologne, Cologne, Germany; 2 Institute of Physiology I, Life and Brain Center, University of Bonn, Bonn, Germany; 3 Department of Physiology, Huazhong University of Science and Technology, Tongji Medical College, Wuhan, China; 4 Department of Molecular and Cellular Sport Medicine, German Sport University, Cologne, Germany; 5 Department of Cardiology, Medical University of Lübeck, Lübeck, Germany; University of Milan, Italy

## Abstract

Dihydropyridines (DHP), which nifedipine is a member of, preferentially block Ca^2+^ channels of different cell types. Moreover, influx of Ca^2+^ through L-type Ca^2+^ channels (LTCCs) activates Ca^2+^ signaling pathways, which in turn contribute to numerous cellular processes. Although LTCCs are expressed in undifferentiated cells, very little is known about its contributions to the transcriptional regulation of mesodermal and cardiac genes. This study aimed to examine the contribution of LTCCs and the effect of nifedipine on the commitment of pluripotent stem cells toward the cardiac lineage *in vitro*. The murine embryonic stem (ES, cell line D3) and induced pluripotent stem (iPS, cell clone 09) cells were differentiated into enhanced green fluorescence protein (EGFP) expressing spontaneously beating cardiomyocytes (CMs). Early treatment of differentiating cells with 10 µM nifedipine led to a significant inhibition of the cardiac mesoderm formation and cardiac lineage commitment as revealed by gene regulation analysis. This was accompanied by the inhibition of spontaneously occurring Ca^2+^ transient and reduction of LTCCs current density (*I*_CaL_) of differentiated CMs. In addition, nifedipine treatment instigated a pronounced delay of the spontaneous beating embryoid body (EB) and led to a poor surface localization of L-type Ca^2+^ channel α_1C_ (Ca_V_1.2) subunits. Contrary late incubation of pluripotent stem cells with nifedipine was without any impact on the differentiation process and did not affect the derived CMs function. Our data indicate that nifedipine blocks the determined path of pluripotent stem cells to cardiomyogenesis by inhibition of mesodermal commitment at early stages of differentiation, thus the proper upkeep Ca^2+^ concentration and pathways are essentially required for cardiac gene expression, differentiation and function.

## Introduction

Embryonic stem (ES) and induced pluripotent stem (iPS) cells are cells able to self-renew and give rise to cells found in all three embryonic germ layers (ectoderm, mesoderm and endoderm) [1]–[3]. Under specified conditions ES cells can differentiate i*n vitro* into spontaneously beating cardiomyocytes (CMs) among other cells types, allowing to study early cardiogenesis [4]–[6]. The induction of pluripotent stem cell differentiation towards cardiac fate is governed by multiple developmental steps such as mesoderm induction and maturation of early cardiomyocytes [7]. Cardiac development is a dynamic process that is tightly orchestrated by the sequential expression of multiple signal transduction proteins and transcription factors working in a combinatory manner [8], and even subtle perturbation of this events can have catastrophic consequences in the form of congenital disease such as heart failure. Entry of cells into the cardiac lineage is dependent upon appropriate external signals coupled to the expression of a set of transcription factors that initiates and activates the network for cardiac gene expression, cardiomyocytes differentiation and maturation [4], [5], [9]. Previous studies mainly focused on the analysis of chemical biology, in which small molecules are identified and used to regulate cell fate or modulate cell reprogramming [10], [11].

Although the mechanisms controlling the temporal aspects of mesoderm induction are poorly understood, both the sarcolemmal LTCCs and Ca^2+^ signaling pathways might play pivotal roles in the specification of mesoderm to the cardiac lineage. The LTCCs, like other membrane channels and receptors, is a heteromultimetric complex, which is also connected with signaling molecules, enzymes, or proteins [12]. Moreover, LTCCs antagonists of the dihydropyridines (DHP) type such as nifedipine and non-DHP, verapamil have been widely used in clinical settings because of their strong antihypertensive effects due to relaxation of vascular smooth muscles [13]. DHPs bind to a site on the α_1_ subunit of the L-type Ca^2+^ channel and prevent Ca^2+^ influx, therefore reduce the cytosolic Ca^2+^ concentration and in turn reduce excitation/contraction coupling.

In adult CMs, the influx of Ca^2+^ through LTCCs is the major boost mechanism for rise of intracellular Ca^2+^ levels. This mechanism is accomplished via activation of the ryanodine receptors, localized in the sarcoplasmic reticulum (SR), which in turn induces Ca^2+^ release from SR [14]. This process further facilitates Ca^2+^ binding to the myofilament regulatory protein, known as troponin C, which then switches on the contractile elements for CMs contraction. In embryonic CMs, mainly transarcolemmal Ca^2+^ influx via LTCCs modulates the intracellular Ca^2+^ concentration [Ca^2+^]_i_ during systole and diastole [15] and the generation of spontaneous cardiac activity [16], [17], because the t-tubule system and the SR, known as Ca^2+^ handling facilitators are structurally and functionally underdeveloped in embryonic CMs [18].

In early developmental stages of embryonic CMs, voltage-gated Ca^2+^ channel subtypes have been shown to allow modulation of intracellular Ca^2+^ signals capable to influence cellular physiology in terms of proliferation, apoptosis, signal transduction and membrane potential [19]–[21]. As one piece of the puzzle, it was demonstrated that disturbances of Ca^2+^ signaling resulted in inhibition of mRNA expression and activity of cardiac-specific genes [22]–[25]. However, little is known about the promotion of ES cells to form committed cardiac mesoderm and factors to give rise to cardiomyocytes. Identifying the characteristic modulation of cardiac gene expression and functional properties associated with LTCCs will provide a mechanistic framework to understand how this channel contributes to cardiac differentiation. We therefore hypothesized that Ca^2+^ influx through LTCCs, especially at a very early stage of cell fate specification and differentiation may be of importance for activation and regulation of the Ca^2+^-dependent cardiac differentiation program.

Using the well established in vitro model system of ES and iPS cells, here we provide by molecular, histochemical and electrophysiological approaches, some preliminary evidence of the mechanism by which LTCCs blockage represses differentiation of mES cell-derived CMs. This study was also conducted to unravel the effects of Ca^2+^ influx as a regulatory key event in cardiac fate determination, providing a significant improvement in our mechanistic understanding of the critical role of Ca^2+^ signals/pathways plays in cardiomyogenesis.

## Methods

### Cell Culture and *in vitro* Differentiation

**Figure 1:**
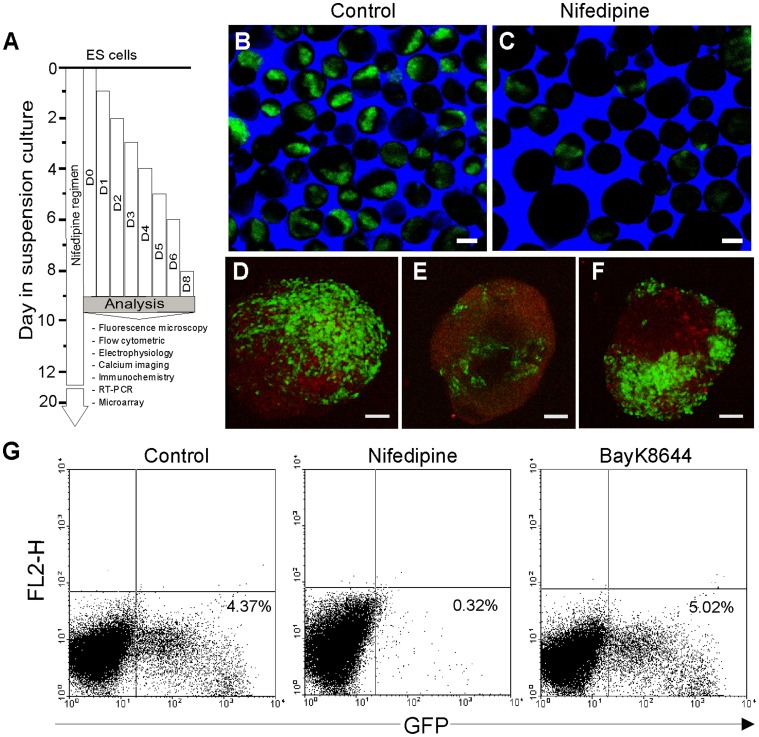
The presence of nifedipine in culture medium inhibits differentiation of ES cell-derived CMs. (***A***) Protocol used to determine the effect of nifedipine application during cell differentiation. Note that for beating EBs and single cell electrophysiology and Ca2+ imaging experiments, analysis was performed at least 24 hours after washout to reflect long-term changes instead of acute drugs effects. (***B–C***) Representative experiments showing EBs with GFP expressing areas cultured under control conditions (*B*) and after nifedipine-treatment (10 µM) (*C*) of ES cells. (***D–F***) Representative EBs showing EGFP+ CMs differentiated of ES cells under control conditions (***D***), after nifedipine- (***E***) and BayK8644- (***F***) treatment. (**G**) Representative FACS analysis of ES cell-derived CMs generated under control, nifedipine- and BayK8644-treated conditions. (Scale bars 50 µm (B, C) and 20 µm (D–F).

We have used the conventional murine ES cell line D3 (clone α-pig44) [26], engineered to express eGFP under control of α-MHC promoter, to specifically detect cardiomyocytes during the developmental process. Cells were maintained in an undifferentiated state by culturing on a monolayer of mitotically inactivated embryonic fibroblast cells feeders in DMEM supplemented with nonessential amino acids (0.1 mM), L-glutamine (2 mM), penicillin, streptomycin (50 µg/ml each), β-mercaptoethanol (0.1 mM), leukemia inhibitory factor (LIF) (500 U/ml), and 15% fetal calf serum (FCS). The ES cell D3 α-pig44 [26] were differentiated into spontaneously beating CMs as reported [27]. Induced pluripotent stem (iPS) cell clone 09, generated from murine embryonic fibroblasts (MEFs) by Wernig and coworkers [28] and as previously described [6], [29], was also used to confirm the initial results obtained from native murine ES cells. The principle of differentiation is schematically illustrated in Figure 1A and described in Methods S1.

### Study Design and Analysis of CMs (eGFP-positive)

For detailed design protocol, please see Supplementary materials. Briefly, saturating concentration of Nifedipine (10 µM), Verapamil (10 µM) or (−) BayK8644 (an activator of the LTCCs, 10 µM) was added to undifferentiated cells (day 0) and after EB formation at different days of culture. Nifedipine was thoroughly washed out 24 hours before analysis.

### Microarray Procedure

Three independent total RNA samples were extracted from EBs at day 4 and beating clusters of EBs at day 12 of differentiation using the TRIzol reagent. The characteristics of these samples are summarized in Table S1.

Biotin-labeled cRNAs were prepared using the Ambion® Illumina RNA amplification kit (Ambion Europe), 1.5 µg was hybridized to Sentrix® whole genome bead chips (Mouse WG-6 v2.0, Illumina) carrying 45281 probe sets and scanned on the Illumina® BeadStation 500x. Data analysis procedure and statistical methods were performed as previously described [30].

### RT-PCR and Quantitative RT-PCR Analysis

For reverse-transcription (RT) polymerase chain reaction (PCR) and real-time PCR analysis, total RNA was isolated from undifferentiated cells (day 0) or EBs at day 2–5, 8 and day 12 contracting areas (CAs) using the RNeasy mini kit (Qiagen GmbH, Germany) or TRIzol (Invitrogen) and analyzed as previously described [30], [31]. Details and primer sequence are depicted in methods S1 and Table S2, respectively. All experiments were conducted in triplicate.

### Flow Cytometry

For flow cytometric (FC) analysis, EBs were washed with PBS and then dissociated to a single cell suspension by trypsin treatment (120 µl of trypsin/EDTA solution) for 2–3 min. Then, 1 ml of DMEM +20% FCS was added to the single-cell suspension. After centrifugation (1000 rpm) for 5 min, cells were resuspended to a concentration of 5 × 10^5^ cells/ml in PBS and then analyzed with the FACScan flow cytometer (Becton Dickinson, USA). The emitted fluorescence of GFP was measured at 530 nm (FITC band pass filter) and analyses were performed using the Win MDI 2.8 software.

### Immunostaining

Immunofluorescence was carried out using standard techniques. Detailed methodology is described in Supplementary materials. For analysis the Zeiss AxioVision 4.5 software package (Zeiss, Göttingen, Germany) was used.

### Electrophysiology

To characterize the electrophysiological properties of ES cell-derived CMs cultured under nifedipine-treated conditions, we used the whole cell configuration of the patch-clamp technique [18], [32]. All recordings were performed using an EPC 9 amplifier and the Pulse acquisition software (Heka Elektronik, Lambrecht, Germany). Detailed protocols and analyses as well as the composition of extra- and intra-cellular solutions used during patch-clamp measurements are provided in Supplementary materials. All substances were, if not stated otherwise, obtained from Sigma-Aldrich.

### Ca^2+^ Imaging

Single-cell Ca^2+^ imaging was performed, using the cell membrane-permeant Ca^2+^ indicator fura-2AM (Molecular Probes, Eugene, USA) according to standard protocols (see Supplementary material). Intracellular Ca^2+^ were measured using an imaging system from TILL-Photonics (TILL Photonics, Planegg, Germany) and the emission data as well as the method used to access sarcoplasmic reticulum (SR) Ca^2+^ content, were analyzed as described in Supplementary materials.

### Statistical Analysis

Data are presented as mean±SEM. Statistical analysis was performed using Student’s *t* test for grouped data (in case of two groups) or one-way analysis of variance (in case of multiple groups). A P-value of <0.05 was considered statistically significant.

## Results

### Cardiac Differentiation of ES and iPS Cells

ES and iPS cells were differentiated to CMs in the presence of DMSO (solvent control), nifedipine, verapamil (LTCCs blockers) or BayK8644 (opener of all L-type channels (Ca_V_1.2 family)). After 7 days in culture, enhanced Green Fluorescent Protein (EGFP)-positive clusters of differentiated CMs could be detected in EBs generated under control conditions (Figure 1B and D) and, to a significant lesser extend, in the continuous presence of nifedipine (Figure 1C and E). However, the presence of the Ca^2+^ channel agonist, BayK8644 in culture did not considerable improve the differentiation of ES cells into CMs (Figure 1F). Most of the EBs cultured under control conditions expressed larger and brighter EGFP-positive areas (video S1) as compared with those obtained under nifedipine treatment (video S2). Florescence microscopy experiment also revealed significant reduction of CMs derived under nifedipine treatment (Figure S1A and S1B). This result was confirmed by the flow cytometric (FC) analysis (Figure 1G), which showed a significant decrease of EGFP-positive cells from 4.4% under control conditions to 0.3% under nifedipine-treatment. However, the percentage of CMs generated under BayK8644 treatment was increased by trend to 5.0% as compared to controls.

**Figure 2:**
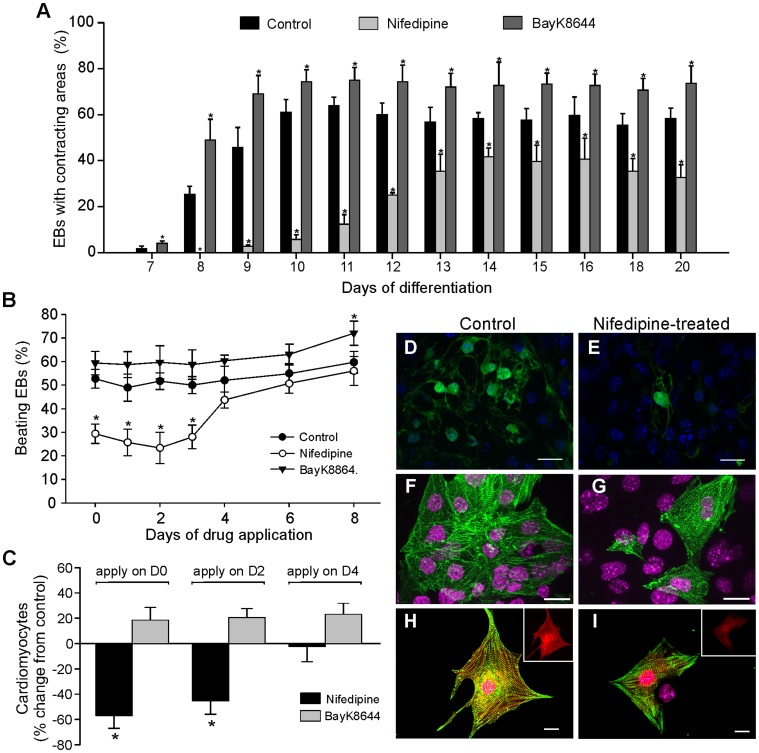
Nifedipine repressed the cardiac differentiation profile. (***A***) Time course of the incidence of ES cell-derived contracting EBs generated in the absence (control) or presence of nifedipine or BayK8644. The mean±SEM of the percentage of EBs with contracting areas during differentiation is depicted. (***B***) Percentage of beatings EBs at day 12 of differentiation derived from ES cells following nifedipine and BayK8644 administration at different times. Note that addition of nifedipine before day 4 of differentiation reduced the percentage of EBs containing beating CMs significantly below both control and BayK8644 levels. (***C***) Percentage of CMs changes from control condition obtained by FACS analysis after 12 days of cell differentiation under nifedipine and BayK8644. Nifedipine and Bayk8644 were applied on days 0 (D0), 2 (D2) and 4 (D4) of differentiation. (***D–E***) Immunofluorescence detection of CMs (green) in EBs after 12 days of differentiation. (***F–G***) Immunostaining of representative dispersed cells from beating clusters of EBs derived under control and nifedipine-treated conditions with anti-sarcomeric α-actinin (red) (1∶800). (***H–I***) Cardiomyocytes isolated from beating areas of EBs at day 12 of differentiation in both control and nifedipine-treated conditions show sarcomeric striations when stained for α-sarcomeric actinin. Insets show the fluorescence intensity of EGFP (red). Hoechst 33342 was used to stain nuclei (blue or magenta). Scale bars: 20 µm.

To determine the development of contracting EBs over time in control and nifedipine-treated cultures, plated EBs were examined daily from day 7 to day 20 of differentiation for spontaneously CAs (Figure 2A). On day 7 of differentiation (2 days after plating), less than 2% of the plated EBs show spontaneously CAs in control cultures. The percentage of EBs contained such CAs continued to rise until 64% at day 11 of differentiation, whereas very few EBs (5, 7%) with spontaneously CAs were observe on day 10 of differentiation in nifedipine-treated cultures. The percentage of EBs with CAs increases slightly and remains low until day 12 of differentiation. After day 12, the percentage continued to increase until 41, 7% of the EBs show CAs on day 14 of differentiation. However, the maximum percentage of EBs with CAs were seen on day 10 (one day before as compared to control cultures) in BayK8644-treated cultures and were remain unchanged and continued to beat vigorously until day 20 (the longest period observed) of differentiation (Figure 2A). EGFP-positive cells were seen to have more synchronized contracting in BayK8644 and control treatment group through real-time imaging of the beating Ebs. This opposite and positive effect of BayK8644 suggests that it may increases Ca^2+^ current in a way that should allow extra Ca^2+^ influx to be used efficiently during cardiomyogenesis.

In addition, to find the exact time point at which nifedipine affects cardiomyogenesis, nifedipine and BayK8644 were added to ES cells at day 0 or EBs at day 1–4, 6, and 8 of differentiation and EBs with spontaneously CAs were examined microscopically during the differentiation. We found that nifedipine only impacted the appearance of CAs within the EBs when applied before day 4 of ES (Figure 2B) and iPS (data not shown) cell differentiation, which goes along with the expression of most markers of early mesoderm commitment. Quantification of the number of CMs by FC analysis at day 14 of differentiation also revealed a significant decreased of CMs to 56.8±10% (*P*<0.01) in nifedipine-treated cultures when added at day 0 and to 40.4±11% (*P*<0.01) at day 2 of differentiation, whereas BayK8644 treatment was without significant effect as compared to control condition (Figure 2C, n = 5). But the addition of nifedipine after day 2 of differentiation did not affect the output of ES cell-derived CMs. The obvious reduction of the number of CMs derived from nifedipine-treated culture was confirmed by α-actinin immunolabeling of single CMs showing a reduction of α-actinin positive cells by approximately 66% (*P*<0.05, Figure S1C). In addition, using another stem cell source, nifedipine also reduced the percentage of beating EBs and CMs derived from reprogrammed iPS cells where as BayK8644 significantly induced CMs differentiation (Figure S1D) as compared to control conditions and to ES cells. These above results suggest the possibility that nifedipine may not only be blocking the process or pathways linking electrical excitation to contraction of CMs, but also inhibited the expression of cardiac-specific genes during cardiomyogenesis.

Moreover, in the presence of BAPTA-AM (an intracellular Ca^2+^ chelator, 10 µM) and EGTA (an extracellular Ca^2+^ chelator, 2 mM), the number of spontaneously beating EBs was significantly decreasing (data not shown). Similar results were obtained in Ca^2+^-free condition, providing strong evidence of the role of both extracellular and intracellular Ca^2+^ sources for cardiac differentiation in mammalian cells.

In contrast, the presence of verapamil, a non-dihydropyridine and LTCCs blocker, during the differentiation process increased significantly the number of contracting EBs derived from ES (Figure S1D) and iPS cells. In addition the number of CMs generated under verapamil was significantly increased (Figure S1E), confirming the disparate effects of both Ca^2+^ channels blockers nifedipine and verapamil as previously mentioned [33].

### Molecular and Structural Characterization of ES cell-derived CMs

To compare the morphological characteristics of the differentiated CMs generated under nifedipine-treated conditions, we performed immunostaining for different cardiac markers. The analysis of EGFP-positive cells confirmed the above mentioned reduction of CMs by nifedipine (Figure 2D–G). Moreover, higher magnification of single CMs revealed the presence of a cross-striated pattern of α-actinin but only in the limited area of nifedipine-treated CMs with high intensity of EGFP (Figure 2H and 2I). To shed light on how nifedipine affects the course of lineage specification and CMs differentiation, the temporal expression patterns of several markers present in EBs at day 4 and beating clusters at day 12 of differentiation was profiled by Illumina BeadStation 500x system. Hierarchical clustering (Figure S2A) and principal component analysis (Figure S2B) showing the expression of variable genes in control and nifedipine-treated cultures are depicted. A closer look of result at differentially expressed genes (*P*<0.05; >2-fold change; difference between mean intensity signals >100) revealed that 47 probe sets (1.03%) at day 4 and 192 probe sets (4.2%) at day 12 of differentiation significantly differ in their expression levels between control and nifedipine-treated cells. Log ratio analysis revealed significant differences (fold change ≥2, *P*<0.05, n = 3) of genes involved in regulation of cardiomyocytes progenitor and development (data not shown), confirming the effect of nifedipine on ES cells during differentiation towards CMs.

**Figure 3:**
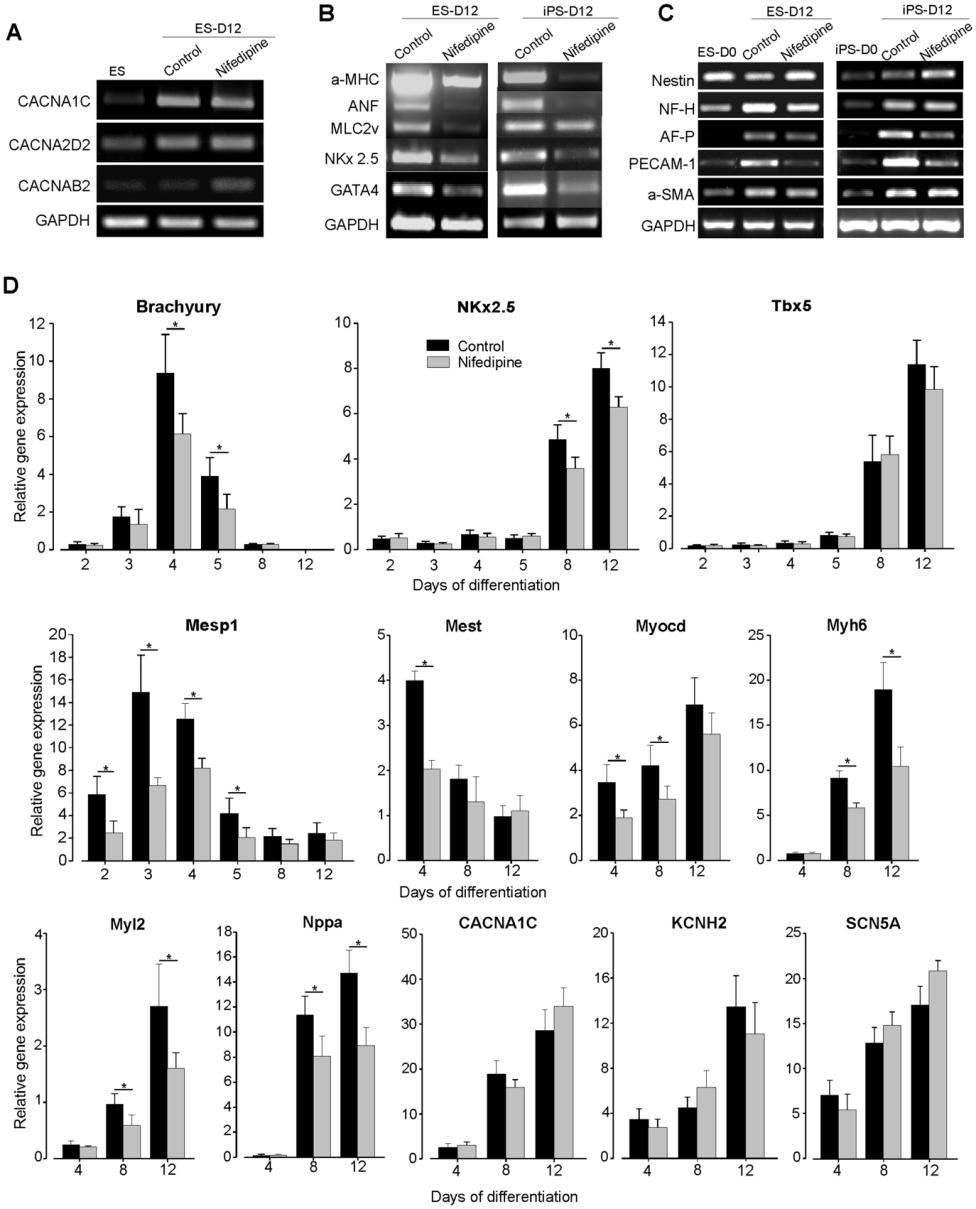
Nifedipine repressed the expression level of most specific cardiac and mesoderm genes. (***A***) RT-PCR for L-type Ca^2+^ channel α-subunit isoforms in undifferentiated ES cells and CMs at day 12 of differentiation, demonstrating (i) presence of L-type Ca^2+^ channel mRNA even in undifferentiated ES cells and (ii) an increase in mRNA encoding the CaV1.2 subunit of the channel. (***B***) RT-PCR analyses of cardiac-specific and transcription factors *α-MHC/Myh6, ANF/Nppa, MLC2v/Myl2, NKx2.5* and *GATA4* in beating cluster at day 12 of both ES and iPS differentiation. (***C***) RT-PCR analyses of representative markers, PECAM-1 (mesoderm, endothelial cell), AFP (endoderm, early hepatocyte progenitor), Nestin and NF-H (ectoderm, neural progenitor) and α-SMA (mesoderm) in ES and iPS beating cluster at day 12 of differentiation. (***D***) Quantitative real-time PCR analyses of mesoderm and cardiac markers and specific transcript (*Brachyury T, Mesp1, NKx2.5, Tbx5, Mest, Myocd, Myh6/α-MHC, Myl2/MLC2v, and Nppa/ANF*), as well as cardiac ionic channels (*CACNA1c, KCNH2* and *SCN5A*) expression at different stage of ES cell differentiation. GAPDH was used as housekeeping gene and served to normalize the result. Results are reported as the means±SEM (n = 3). *P<0.05 vs. control CMs.

To gain more insight on the effect of nifedipine, we afterwards performed RT-PCR to confirm our microarrays results. As shown in Figure 3A, LTCCs subunit genes *CACNA1C (α_1c_), CACNAB2 (β_2_) and CACNA2D2 (α_2_δ_2_)* were present already in undifferentiated ES cells (conditioned medium, feeder-free) with higher expression at day 12. We also found that the cardiac markers *α-MHC* or *Myh6, ANF or Nppa, MLC2v* or *Myl2, GATA4* and *Nkx2.5* were lower expressed in cells generated under nifedipine-treated cultures at day 12 of ES and iPS differentiation (Figure 3B). In addition, we examined the mRNA expression level of AFP (endoderm, early hepatocyte progenitor), Nestin and NF-H (ectoderm, neural progenitor) of cell generated under nifedipine. For both ES and iPS cells, only a weak decrease of NF-H expression level was observed in nifedipine-treated cells (Figure 3C) whereas Nestin and AFP expression levels were unchanged. Moreover, in both ES and iPS cells, significant decrease of PECAM-1 (mesoderm, endothelial cell) expression level was observed in nifedipine-treated condition as compare to control. The analysis of α-smooth muscle actins (α-SMA), another mesodermal sub lineage, revealed no significant effect on the mRNA expression level of both cell lines. These results indicates the presence of other differentiated cell types along with cardiomyocytes and confirming, at least in part, the specific action of nifedipine on cardiac precursor cells.

To elucidate stage-dependent effects of nifedipine and to provide a quantitative assessment of gene expression during the differentiation of murine ES cell toward cardiac lineages, we further performed quantitative RT-PCR analysis (Figure 3D). The primitive streak and cardiomesoderm markers *TBra, Mest* and *Mesp 1* showed their peak expression at day 3 or 4 of differentiation and were strongly downregulated in the nifedipine-treated group. We also detected a significant down-regulation of the cardiac transcription factor *Myocd* at day 4 and 8. Additionally, a significant repression of cardiac-specific structural genes *Myh6*, *Myl2* and *Nppa* was observed in the nifedipine group, confirming our RT-PCR results. No effects of nifedipine treatment were observed on the genes expression level of ions channels studies such as *CACNA1C*, *KCNH2* and *SCN5A*. As the differentiation is generally associated with changes in proliferation rates of the cells, nifedipine-treatment did not reveal significant changes in the proliferation capacity of cells (Figure S2C) as show by CellTiter-Glo assay. Further, the apoptosis was evaluated with Annexin V staining. This analysis revealed no apoptotic induction after day 12 of nifedipine treatment in comparison to control cultures (Figure S2D).

### Electrophysiology - APs and Hormonal Regulation

**Figure 4:**
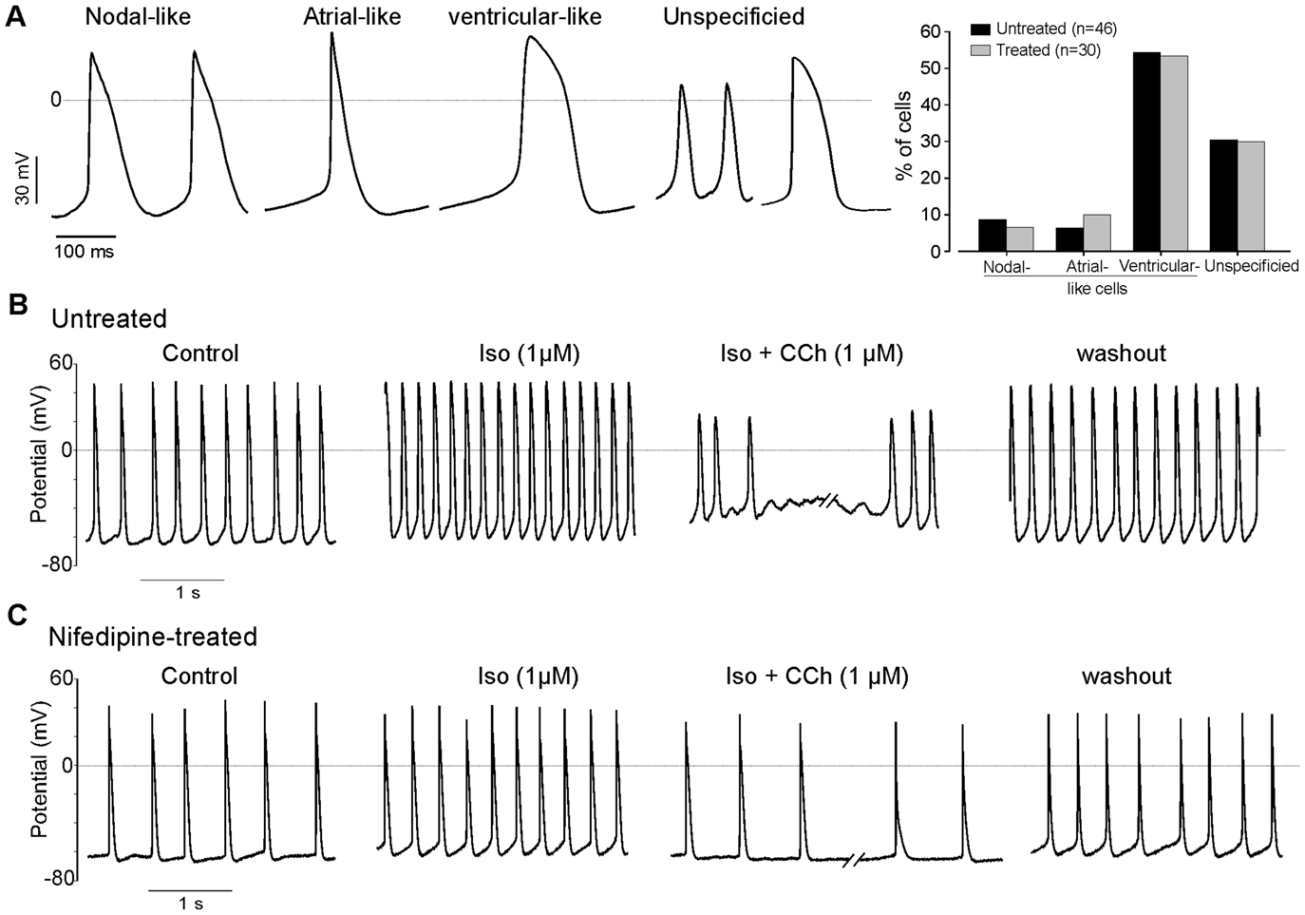
Nifedipine does not affect the generation of specific cardiac subtypes as well as the β-adrenergic and muscarinic regulation pathways. (***A***) Representative AP recordings from spontaneously beating ES cell-derived CMs revealed differentiation of nifedipine-treated cell into the different cardiac subtypes: nodal-, embryonic atrial- and embryonic ventricular-like CMs, note the presence of unspecified CMs (left panel). Statistic analysis (right panel) of different cardiac subtypes generated under control and nifedipine-treated cultures. (***B–C***) Representative AP of untreated (*B*) and nifedipine-treated (*C*) ES cells-derived CMs showed a prominent positive chronotropic effect of Iso (1 µM) (middle left) and negative chronotropic response to CCh (1 µM) (middle right) application. These effects could be partially reversed by washout. The dotted lines indicate the zero current level.

To test whether loss of gene expression actually reflects CMs with distinct functional properties, we performed patch-clamp measurement to record spontaneous action potentials (APs). In control CMs (n = 32) and in CMs derived from nifedipine-treated group (n = 21) we could measured APs of all 3 major cardiomyocytes subtypes. These could be classified as nodal-like (9.3% vs. 9.5%), embryonic atrial-like (12.50% vs. 14.2%) and ventricular-like (78.1% vs. 76.2%) APs (Figure 4A, left), respectively based on their shape and properties as described previously [19], [34] and summarized in Table 1. However, some CMs in the control (n = 14) and nifedipine-treated (n = 9) groups did not match any of the cardiac subtype and were classified as unspecified. The percentages of different AP subtypes recorded in CMs derived from the control and nifedipine treatment group were similar (Figure 4A, right). The presence of nifedipine during the differentiation process led to a significant alteration of AP parameters such as beating frequency, amplitude and AP duration at 90% of repolarization (Table 1).

**Table 1:** AP parameters of CMs obtained from EBs generated under control and nifedipine-treated conditions.

Cell type	Condition	n	Frequency (Hz)	Amplitude (mV)	APD50 (ms)	APD90 (ms)	dV/dt max (V/s)	MDP (mV)
**Atrial-like**	Control	3	6.8±0.9	53.3±0.7	26.6±1.8	42.8±2.2	7.0±1.2	−54.6±2.2
	Nifedipine	2	3.2±1.0*	48.5±1.7*	33.5±1.4*	52.3±3.5*	8.6±1.3	−55.4±1.7
**Nodal-like**	Control	4	5.4±0.8	47.1±3.5	34.3±5.0	48.4±5.0	4.9±1.2	−51.7±3.7
	Nifedipine	3	3.6±0.9*	40.8±1.0*	41.0±2.6	57.1±1.7*	5.5±0.6	−50.1±0.8
**Ventricular-like**	Control	25	3.8±0.8	57.0±3.7	47.9±8.0	57.3±8.5	12.3±2.4	−58.0±5.2
	Nifedipine	16	2.6±1.0*	50.9±6.8	54.4±7.6	66.5±7.5*	11.8±2.5	−59.4±2.6

**Abbreviations:** n, indicates the cell number; APD50/APD90, AP duration measured at 50% or 90% of repolarization; dV/dtmax, maximum rate of rise of AP; and MDP, maximum diastolic potential.

* P<0.05 compared with control condition.

As critical determinant of normal CM function is the intact response to hormones and transmitters of the central nervous system, we also assessed the functional integrity of the cells. In ES cell-derived CMs generated under control (Figure 4B) and nifedipine-treated (Figure 4C) conditions, the application of β-adrenergic agonist Iso (1 µM) and acetylcholine analog CCh (1 µM) receptor stimulation on AP frequency induced a significant positive and negative chronotropic effects, respectively. Isoprenaline induced additionally a comparable increase of the AP frequency whereas, subsequent application of CCh during the same measurements was able to inhibit the effect of Iso, indicating the presence of coupled β-adrenergic and muscarinic signaling cascades in CMS generated under nifedipine treatment.

### Ion Channel Characterization

For a detailed analysis of the functional expression of the main voltage-gated ion channels, which control the electrical impulses in the heart and to evaluate the side effect of nifedipine, whole-cell voltage-clamp experiments were performed in cells generated under nifedipine at day 4 and 12 of ES cells differentiation.

**Figure 5:**
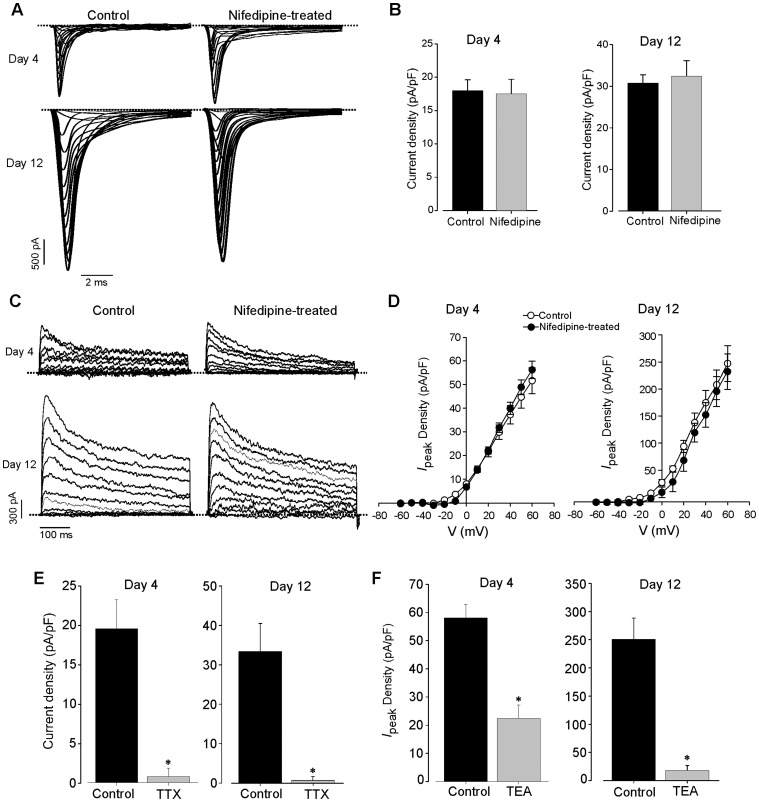
Functional characteristics of Na^+^ channel and depolarization-activated outward K^+^ currents. (***A–B***) Representative traces (A) and currents densities (B) of Na^+^ current recorded from a control and nifedipine-treated cells at days 4 (non-beating cell) and 12 (beating CM) of differentiation. (***C–D***) Representative traces and current-voltage relationships (*I/V*) of the peak depolarization-activated outward K^+^ current (*I*_peak_). (***E–F***) Effect of acute application of Na^+^ channel (*E*) blocker TTX (1 µM) and non-specific K^+^ channel (*F*) blocker TEA (10 mM). The dotted lines indicate the zero current level.

*I*_Na_ was not different between both untreated and nifedipine-treated groups (original traces, Figure 5A). Also *I*_Na_ density (Figure 5B) was similar (*P*>0.05) between both groups at day 4 (18.1±1.6 pA/pF, n = 12 *versus* 17.5±2.2 pA/pF, n = 18) and 12 (30.7±2.0 pA/pF, n = 9 *versus* 32.4±3.7 pA/pF, n = 7) of differentiation.

We further characterized the expression and function of depolarization-activated outward K^+^ currents. Peak currents (*I*_peak_) and late currents (*I*_sus_) were measured as described in methods S1 [35]. Both groups of CMs showed depolarization-activated outward K^+^ current (Figure 5C) at day 4 and 12. The current-voltage relationship (*I/V* curves) of *I*_peak_ (Figure 5D) and *I*_sus_ (data not shown) was not significantly different. At both day 4 and 12 of differentiation, *I*_peak_ and *I*_sus_ densities of cells generated under nifedipine were similar to control groups. Control experiments to tests for intact pharmacology of *I*_Na_ and *I*_k_ of CMs generated in the presence of nifedipine revealed no acute effect of nifedipine on *I*_Na_ density an at maximum outward *I*_k._ However TTX (1 µM), a powerful and specific voltage-gated Na^+^ channel blocker and TEA (10 mM), a non-selective K^+^ channel blocker significantly reduced current density of *I*_Na_ (Figure 5E) and the maximum amplitude of *I*_k_ (Figure 5F), respectively. This result suggests the full functional expression of both *I*_Na_ and *I*_k_ in remaining CMs.

**Figure 6:**
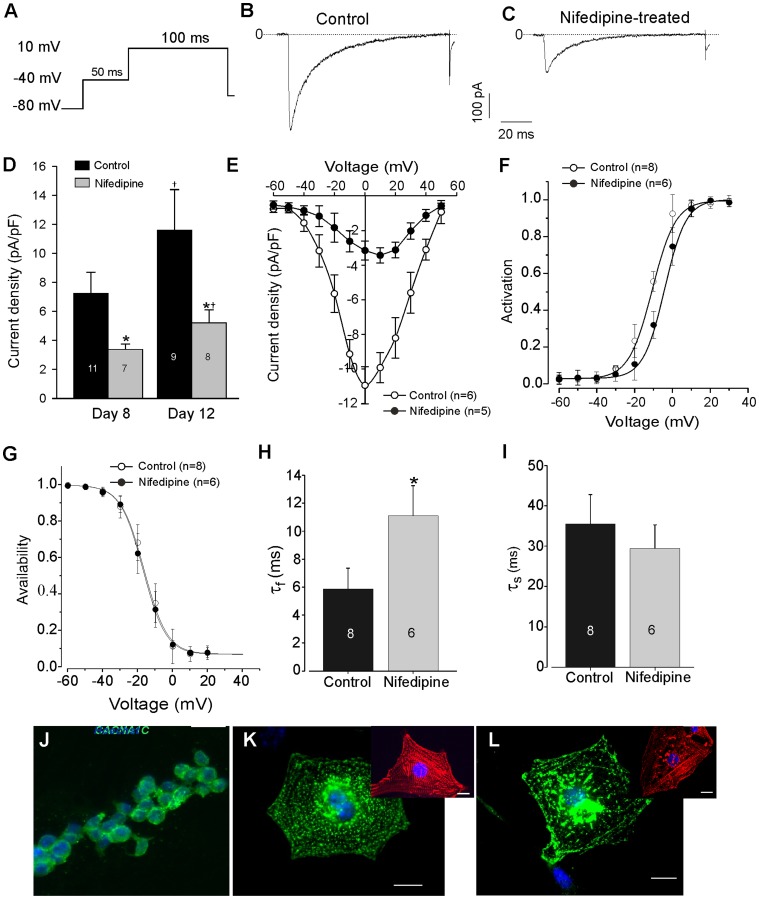
The presence of nifedipine in differentiation medium altered the cardiac *I*_CaL_ density of ES cell-derived CMs. (***A–C***), Representative L-type Ca^2+^ current traces recorded as indicated in protocol (*A*) from CM cultured in control (*B*) and nifedipine-treated (*C*) conditions. (***D***) Current densities recorded at the maximal peak of the current (10 mV) of cells generated in control and under nifedipine-treatment at days 8 and 12 of differentiation. (***E–F***) Current-voltage relationships (*E*) and activation curves (*F*) of *I*_CaL_ recorded in control (open symbols) and nifedipine-treated (filled symbols) CMs. (***G***), Steady-state inactivation curves of control (open symbols) and nifedipine-treated (filled symbols) CMs. (***H–I***) shows the time constants of fast τ_f_ (*H*) and slow τ_s_ (*I*) phases of *I*_CaL_ inactivation recorded in ES cell-derived CMs from control and nifedipine-treated cultures. (***J***) Immunolocalization of L-type calcium Ca_V_1.2 subunit (α_1C_) in undifferentiated ES cells suggests enrichment in the cell periphery and cellular membrane. (***K–L***) Immunocytochemistry of L-type calcium Ca_V_1.2 subunit (α_1C_) in CMs generated under both control (*K*) and nifedipine-treated (*L*) conditions. Insert are positive control experiments from the same bath of cells performed with mouse anti-α actinin. Hoechst 33342 was used to stain nuclei (blue). (Scale bars: 20 µm). * denote significant differences to control and **^†^** significant differences to day 12 of differentiation.

As, the expression level of voltage-gated Ca^2+^ channels at the plasma membrane is a key regulator of Ca^2+^ homeostasis in excitable cells, and of downstream effects such as calcium-dependent transcription, we next studied the biophysical characteristics of *I*_CaL_. Whole cell *I*_CaL_ of CMs was assessed using protocol illustrate in Figure 6A. As shown by representative recording traces (Figure 6B and 6C) and confirmed by the statistic analysis (Figure 6D), *I*_CaL_ was found to be markedly reduced from 11.6±2.8 pA/pF (n = 9) in control CMs to 5.2±0.69 pA/pF (n = 8, *P*<0.01) in CMs generated under nifedipine treatment at day 12 of differentiation. As also shown in Figure 6D, we found an increase of *I*_CaL_ density during differentiation with significance difference between untreated and nifedipine-treated CMs at day 8 (7.2±1.4 vs 3.4±1.1 pA/pF, *P*<0.01) and 12 (11.6±2.8 vs 5.2±0.6 pA/pF, *P*<0.01) of differentiation, suggesting the maturation of *I*_CaL_ density during differentiation. Additionally, the mean *I/V* relationship at the maximum of *I*_CaL_ was decreased by approximately 75% and shifted the maximum peak of *I/V* curve from 0 mV to +10 mV (Figure 6E) of CMs at day 12 of differentiation, suggesting at least in part a phosphorylation of the α_1C_ subunit of the LTCC in CMs generated under nifedipine. To evaluate any alteration of the activation/inactivation process of *I*_CaL_, steady-state activation and inactivation were recorded and calculated as described in Supplemental material online. The half-activation voltage (V_½_) was more positive in CMs from nifedipine group (−6.9±0.6 mV, n = 5 *versus* –11.5±0.5 mV, n = 6; *P*<0.01, Figure 6F). The slope factor (*k*) (5.4±0.4 mV, n = 5 *versus* 6.4±0.5 mV, n = 6; *P*>0.05) as well as the channel availability (steady-state inactivation curve, Figure 6G) were not significantly different. The analysis of the time constant of the fast (τ_f_) and slow (τ_s_) phase of *I*_CaL_ inactivation revealed that τ_f_ (Figure 6H) was clearly faster in control (5.9±1.5 ms at 0 mV, n = 8) than in nifedipine-treated CMs (11.08±2.1 ms at 10 mV, n = 6) (*P*<0.05). However, nifedipine treatment did not significantly affect τ_s_ (Figure 6I). Immunolocalization of LTCCs α_1C_ subunit revealed the expression of this protein already in undifferentiated ES cells (day 0) (Figure 6J). In addition immunostaining of the LTCCs α_1C_ subunit (Ca_V_1.2) in CMs derived from untreated (Figure 6K) and nifedipine-treated (Figure 6L) groups also showed an expression of Ca_V_1.2. However, untreated CMs revealed more homogeneous distribution pattern and presumably higher expression of Ca_V_1.2 in comparison to CMs derived from nifedipine-treated cultures, suggesting that, nifedipine-treatment may result in mislocalization of Cav1.2, causing inefficient excitation-contraction of CMs.

### Intracellular Ca^2+^ Homeostasis

**Figure 7:**
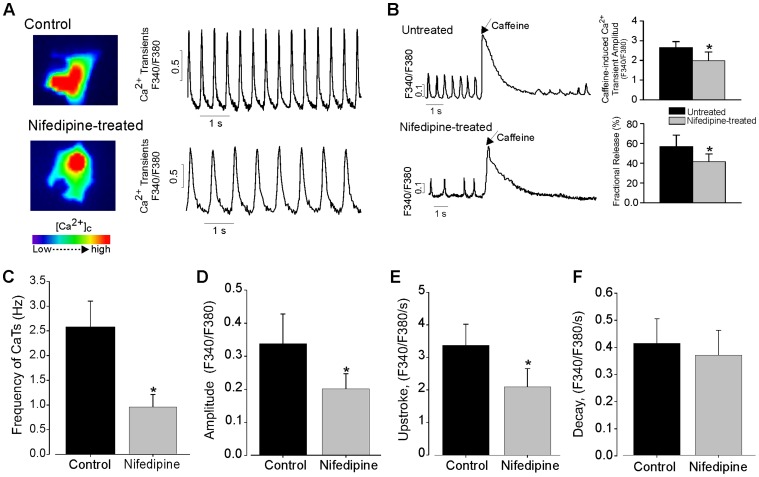
Nifedipine reduced spontaneous Ca^2+^ transient activity in ES cell-derived CMs. (***A***) Representative tracings of spontaneous Ca^2+^ transients recorded in spontaneously beating CMs from control and nifedipine-treated cultures. Fluorescence images (labeled with Fura 2 AM) of the recorded cells are shown on the left panel. (***B***) Left, spontaneous Ca^2+^ transients recorded from CMs derived under untreated (top) and nifedipine-treated (bottom) condition before and after caffeine application. Right, caffeine-induced peak amplitude (top, right) of the Ca^2+^ signals and Fractional release (bottom, right) calculated as the ratio of peak Ca^2+^ concentration under control condition to peak Ca^2+^ concentration induced by caffeine. (***C–F***) Comparison of the frequency (*C*), amplitude (*D*), maximum upstroke (*E*) and decay (*F*) velocity of spontaneous Ca^2+^ transients on day 12 in controls (*n* = 10) and nifedipine-treated *(n* = 8) cultures.

Since Ca^2+^ transients are important events in the regulation of contraction and relaxation of CMs, we, therefore, determine the consequences on overall Ca^2+^ homeostasis by investigating the Ca^2+^ transients of single CMs at different time points of differentiation. Spontaneously evoked Ca^2+^ transients of shorter amplitude were detected in CMs generated under nifedipine-treatment at day 12 of differentiation (Figure 7A). To determine whether the reduction of Ca^2+^ transients in CMs generated under nifedipine is due to reduced SR Ca^2+^ stores load that release Ca^2+^ via functional RyRs or due to reduced LTTC currents, we measured caffeine mobilization of store Ca^2+^ and its effect on [Ca^2+^]_i_ transients by applying caffeine (10 mM,) on to flura-2 AM loaded CMs. As can be observed in Figure 7B, caffeine application elicited an instantaneous, rapid, and large release of Ca^2+^ from the intracellular stores, resulting in a high amplitude caffeine-induced Ca^2+^ transient in control (n = 11) as well as in nifedipine-treated (n = 7) CMs (*P*<0.05). The maximum amplitude of caffeine-induced Ca^2+^ transient (indicating the SR Ca^2+^ content was significantly reduce in CMs generated under nifedipine-treated compared to untreated (Figure 7B, right top), which could fully explain the decrease of *I*_CaL_ density in nifedipine-treated CMs. Interestingly, a significantly lower fractional release of Ca^2+^ was observed in nifedipine-treated CMs compared to untreated CMs, suggesting the presence of functional and less SR Ca^2+^ stores load in nifedipine-treated CMs and supporting a role for nifedipine-sensitive receptors in LTCCs-mediated Ca^2+^ influx. As also revealed by the spontaneous AP measurements, the frequency of Ca^2+^ transients was significantly decreased (0.95±0.3 Hz, n = 9 *vs.* 2.6±0.5 Hz, n = 12, *P*<0.01, Figure 7C) as well as the relative amplitude (0.34±0.09, n = 9 *vs.* 0.2±0.04, n = 12, *P*<0.01, Figure 7D) in the nifedipine group. Moreover the maximum upstroke velocity (*V*_max_, Figure 7E) of the Ca^2+^ transients was decreased by approximately 70% (Figure 7F). The Ca^2+^ transients changed time-dependingly during differentiation and displayed large differences in amplitude and frequency in both untreated and nifedipine-treated cells (Figure S3A).

Addition of nifedipine into the culture medium after 2 days of differentiation had not significantly impact on the Ca^2+^ transients (Figure S3B), which were still sensitive to acute application of nifedipine (Figure S3C and S3D).

These data indicate that nifedipine reduces the expression of Ca_v_1.2, impairing the intracellular cardiac transient, and that LTCCs is necessary to maintain normal Ca^2+^ signaling in the heart.

## Discussion

One of the major challenges of using pluripotent stem cell-based CMs for therapy is the understanding of the mechanisms that control stem cell potential and the development of robust methods to efficiently control their fate. In the present study we demonstrate that LTCCs are *per se* of major importance for an intact cardiac differentiation program. Turbulences of LTCCs function especially during the earliest steps of stem cells differentiation markedly reduce cardiac differentiation and significantly affect electrophysiological properties of the remaining CMs.

Previous studies could prove that LTCCs are already expressed in CMs derived from early developmental stages [15], [22], [36]. These studies hypothesized that in early embryonic CMs mainly transsarcolemmal Ca^2+^ cycling via LTCCs and Na^+^/Ca^2+^ exchanger maintain intracellular Ca^2+^ homeostasis [15], [37]. However, although intracellular Ca^2+^ concentrations have been assumed to be involved in gene regulation and transcription differentiation levels it remained unclear whether LTCCs are expressed in undifferentiated cells and whether a modulation of LTCCs at this stage might affect cardiac commitment. In contrast, most other ion channels are not expressed in the pluri- and multipotent cells that give rise to CMs, but rather appear later during differentiation and/or maturation [15], [37].

In the present study we could now demonstrate that LTCCs are expressed already in undifferentiated murine ES cells. Thus, one might assume that the early expression of LTCCs may be involved in maintenance and modulation of intracellular Ca^2+^ concentrations and might therefore affect Ca^2+^ dependent differentiation processes. As indirect prove for importance of LTCCs-mediated modulation of intracellular Ca^2+^ levels we found that LTCCs blockage by nifedipine significantly decreased the expression level of important mesodermal markers, early and late cardiac markers such as *TBra, Mest, Mesp1, Myocd*, *NKX2.5*, *Gata4, Myh6*, *Myl2* and *Nppa,* suggesting a partial impairment in mesodermal specification and CMs differentiation of ES and iPS cells. Some of these genes have been shown to co-operate with each other to establish a complex system and network, and start the cardiogenic program through elaborate regulation of their downstream cardiac genes [38]. For instance, *Mesp1* is thought to initiate an epithelial-mesenchyme transition in the epiblast and to bind directly to regulatory DNA sequences in the promoters of many members of the important cardiac regulatory network, such as Nkx2.5 thereby promoting development of mesoderm precursors of the cardiac lineage as well as repressing the expression of key genes regulating other early mesoderm derivatives [7]. In contrast, the presence of verapamil, another dihydropyridines LTCCs blocker, increased the number of contracting EBs and the differentiation of ES and iPS into CMs, confirming the previous reports [39]. The dramatic and opposites differences observed between both drugs may be due to different binding sites of DHP and non-DHP on the LTCCs as previously described [1], which may also vary among cell lines and sources with respect to their developmental stage. Moreover, the effect observed could also be attributed to the inhibition of gene expression working in combination manner.

As an important determinant, the time of nifedipine addition was critical for the expression of genes listed above and for cardiac induction. A prominent decrease of cardiac-specific genes and of CMs differentiation was observed when applying nifedipine from day 0 to day 3, time of ES and iPS cell fate specification, but there was not significant effect when nifedipine was applied after day 3 of differentiation. The first striking consequence of the deficiency in the cardiac genetic program is the severe delay in the contractile activity of EBs derived under nifedipine treatment. This may be attributed to decrease in both the number and the function of CMs within the EBs, suggesting the possibility of direct or indirect interaction of nifedipine with regulators involved in cardiac lineage either by blocking the activities of LTCCs or via non-Ca^2+^-channel-dependent mechanism. As revealed in different cellular systems, Ca^2+^ signals of different spatial and temporal properties may activate transcription factors and the signaling cascades involved in their regulation [21], [40].

In the remaining CMs derived from nifedipine-treated group, the cell functions were altered, i.e. reduction of AP and Ca^2+^ transient amplitudes and kinetics. In line with our hypothesis that the functional sarcolemmal expression of LTCCs might have been reduced either due to a reduction of channel protein production and/or surface localization, we found (i) an heterogeneous sarcolemmal surface expression of the α_1C_ subunit, (ii) a significant reduction of *I*_CaL_ density at late developmental stages, and (iii) a prolongation of fast inactivation time constant of *I*_CaL_. The inhibition of *I*_CaL_ density is possibly mediated by downregulation of surface expression of the channels by nifedipine.

In fact, activation of LTCCs can lead to the activation of the transcription factors such as CRE-binding protein CREB, GATA-4, Nkx2.5 MEF and NFAT [41]-[43]. Moreover, calcineurin, PKC, and AKAP150 has been show to form a signaling module that regulates local Ca^2+^ influx in different cell types, but the exact molecular details that underlie this regulation are still unclear. Previous studies have shown that Ca^2+^ entering through LTCCs can diffuse to the cell nucleus and activate nuclear Ca^2+^-dependent enzymes [44], such as Ca^2+^-calmodulin-dependent protein kinase II (CaMKII) [45], which decodes changes in [Ca^2+^]_i_ into corresponding levels of kinase activity and regulate the activity of transcription factors and co-regulators [46]. Thus, an intracellular site action of nifedipine on CaM complex in CMs during differentiation cannot be excluded.

In addition, impaired Ca^2+^ signalling is caused by altered expression of Ca^2+^-depending proteins, among these is the reduced LTCCs expression, activity and density [47]. As LTCC protein (*Cacna1c*) expression was apparently normal in the remaining CMs generated under nifedipine-treatment, the alteration of function is presumably explained by deficits in [Ca^2+^]_i_ and/or Ca^2+^ sparklets activity due to the inhibition of Ca^2+^ influx via LTCCs [12]. We also found that the number of contacting EBs was significantly decreased in (nominally) Ca^2+^-free medium, in the presence of Ca^2+^ chelators BAPTA and EGTA, confirming that Ca^2+^ ions are at least partially involved in maintenance of normal differentiation process of ES cells toward CMs in vitro. This observation is in accordance to previous studies demonstrating that intracellular Ca^2+^ level play an important role ahead in embryogenesis in the specification and commitment steps of the cardiac precursor cell population [22].

As additional prove for an alteration of the transcription process by LTCCs blockage, we found cell proliferation and apoptosis unaltered. Likewise, previous studies demonstrated that nifedipine inhibits rather than induces cell apoptosis of CMs [48]. Interestingly, BayK8644 only showed a tendency to increase the yield of CMs from ES cells at any differentiation stage which was more pronounced in iPS cells, suggesting that single LTCCs activity may be already near its maximum. However, it is unclear whether our observations reflect general differences between ES and iPS cells. The observed differences of the effect of BayK8644 between the analyzed ES and iPS cell clones may not only reflect cell lines or clone differences but may also be due to the pre-selection of the transgenic ES cell line for both robust in vitro proliferation and efficient cardiac differentiations [6].

The finding of remaining but functionally altered CMs obtained despite LTCCs blockage could be attributed either to the combined input of low transcription factors, which might provide sufficient information to activate the cardiac program, or to a combined unknown compensatory mechanism [49]-[51]. The addition of nifedipine might also inhibit Inositol 1,4,5-trisphosphate (IP3) receptors which leads to a partial decrease in the [Ca^2+^]_i_ [5] or would negatively modulate the Ca^2+^-activated K channels which has been shown to drives the fate of pluripotent cells toward cardiomyocytes specification [19].

To exclude unspecific side effects of nifedipine on other channels such as Na^+^ and several types of K^+^ channels that had previously been reported [52]-[54], control experiments were performed which revealed no effects of nifedipine on Na^+^ and K^+^ currents at early and late stages of differentiation. The significant increase of both Na^+^ and K^+^ current densities from day 4 to 12 in CMs generated under both culture conditions reflects an ongoing maturation process.

Despite the clear expression of LTCCs protein in undifferentiated ES cells, we were not able to measure stable *I*_CaL_ in whole cell patch clamp experiments. We assume that the minor expression level of LTCCs in early stage of differentiated cells and the presumably small current flow at that level of differentiation may account for these findings. To further address the functional roles of ion channels in ES or iPS cells, specific knockdown of Ca^2+^ channels with interfering RNA during differentiation into CMs could be used in future studies.

Considering the complexity of the mechanisms (e.g. genes interactions) involved in stem cell differentiation, to attribute these impacts (inhibition of mesodermal gene expression, reduction of the functional surface expression of LTCCs and inhibition of its electrophysiological properties) solely to nifedipine or LTCCs activities would be an exaggeration. A potential contribution of some Ca^2+^ influx pathways such as the Na^+^/Ca^2+^ exchanger shown to be highly expressed, functionally active, and working in the forward and the reverse mode in early stage CMs, to the uphold of the cardiac differentiation program, cannot be excluded [55]. Moreover, caution should to be taken when using pharmacological agents to define channel subtypes in stem cell models. Compounds that have been described as ‘specific’ should be considered ‘selective’ and conclusions regarding ion channels expression profile and function during cell specification need to be profoundly investigated.

These results raise the possibility that in the earliest stages of cardiac differentiation *I*_CaL_ allows sufficient Ca^2+^ entry to induce a cardiac directed differentiation program. This study led us to identify a not previously described critical importance function of LTCCs and nifedipine effect at the level of mesoderm specification for the establishment of the cardiac lineage using ES or iPS cells. These informations could provide insights into the signaling pathways involving in the establishment of the cardiac lineage from human ES/iPS cells.

## Supporting Information

Figure S1 **(A) Representative eGFP+ CMs obtained from dissociated EBs cultured under control conditions or in the presence of nifedipine or BayK8644.** (**B**) eGFP-positive cells (CMs) at day 12 of differentiation generated under nifedipine- and BayK8644-treatment. CMs were manually counted and are expressed as a percentage of total CMs generated under control condition (**C**) The yield of CMs generated under control and nifedipine-treated conditions. Beating EBs were dissected, enzymatically dissociated and cultured on fibronectin- or gelatine-coated plates. Thereafter, cells were staining with nuclei dye Hoechst 33342 and CMs were investigated for α-actinin by means of immunocytochemistry. Alpha-actinin positive cells are represented as a percentage of total Hoechst 33432-labelled (positive nuclei) cells. (**D**)Time course of the incidence of ES and iPS cell-derived contracting EBs generated in the absence (control) or presence of nifedipine, verapamil or BayK8644. The mean±SEM of the percentage of EBs with contracting areas during differentiation is depicted. (**E**) Percentage of CMs (expressed as total of control) obtained by FACS analysis at day 12 of differentiation. Results are reported as the means±SEM (n = 3). * denote significant differences to control. Scale bars: 20 µm.
(TIF)

Figure S2 **(A) Hierarchical clustering and (B) Principal component analysis of variable genes expression during induction of CMs under control and nifedipine-treated conditions at day 4 and 12 of differentiation.** (**C–D**) Proliferation and viability assay of untreated and nifedipine-treated ES cells. Nifedipine did not alter cell proliferation (C) or viability (D) measured after 4 and 12 days of differentiation. A total of 3 independent experiments were studied for each time point (Scale bar, D: 50 µm and C: 200 µm).
(TIF)

Figure S3 **Representative Ca^2+^ transients obtained from control and nifedipine-treated CMs.** (**A)** Developmental changes of spontaneous Ca^2+^ transients of cells generated from EBs at day 2, 4, 6, 8, and 10 of differentiation. Note the time-dependent increase of the Ca^2+^ transients’ amplitude of cell generated under control and nifedipine-treated conditions. (**B)** Effect of nifedipine on spontaneaous Ca^2+^ transients during differentiation. 10 µM Nifedipine was applied at days 0, 2, 4, 6, 8, and 10 during the differentiation process. Thereafter cells were isolated on day 11 or 12 and measured 24 to 48 h later by Ca^2+^ imaging. (**C, D)** Acute effect of nifedipine on spontaneous Ca^2+^ transients. Representative tracings of spontaneous Ca^2+^ transients in untreated (C) and nifedipine-treated (D) cells before (control) and after application of nifedipine (+nifedipine).
(TIF)

Table S1 **List of samples used for global gene expression profiling.**
(TIF)

Table S2 **Primer sequences used for normal and quantitative RT-PCR analysis of total RNA isolated from ES cell-derived CMs cultured under control and nifedipine-treated conditions. Abbreviations:** *ANF/Nppa*, natriuretic peptide precursor A; *ES cell*, embryonic stem cell*; GAPDH,* glyceraldehyde-3-phosphate dehydrogenase; α*-MHC/Myh6*, myosin heavy chain 6, *MLC2v*/*Myl2*, myosin light chain 2 ventricular transcripts; *Myocd*, myocardin; *Mest*, mesoderm specific transcript; *Mesp1,* mesoderm posterior 1; *CACNA1C*, calcium channel, voltage-dependent, L type, alpha 1C subunit; *KCNH2*, potassium voltage-gated channel subfamily H member 2; *SCN5A*, sodium channel, voltage-gated, type V, alpha subunit; *PECAM-1*, platelet/endothelial cell adhesion molecule 1; *AFP,* alpha fetoprotein; *NF-H*, neurofilament H; *aSMA*, alpha-smooth muscle actin.
(TIF)

Methods S1 (DOC)

Video S1 **Representative EB showing EGFP-positive CMs differentiated under control conditions.**
(MPG)

Video S2 **After nifedipine treatment. The videos show the 3D structure of the entire EB.**
(MPG)
