# Supplementary figures and images for: The L-type Ca2+ Channels Blocker Nifedipine Represses Mesodermal Fate Determination in Murine Embryonic Stem Cells
Source: PLoS One. 2013 Jan 8;8(1):e53407. doi: 10.1371/journal.pone.0053407 (PMC3539992; doi:10.1371/journal.pone.0053407)

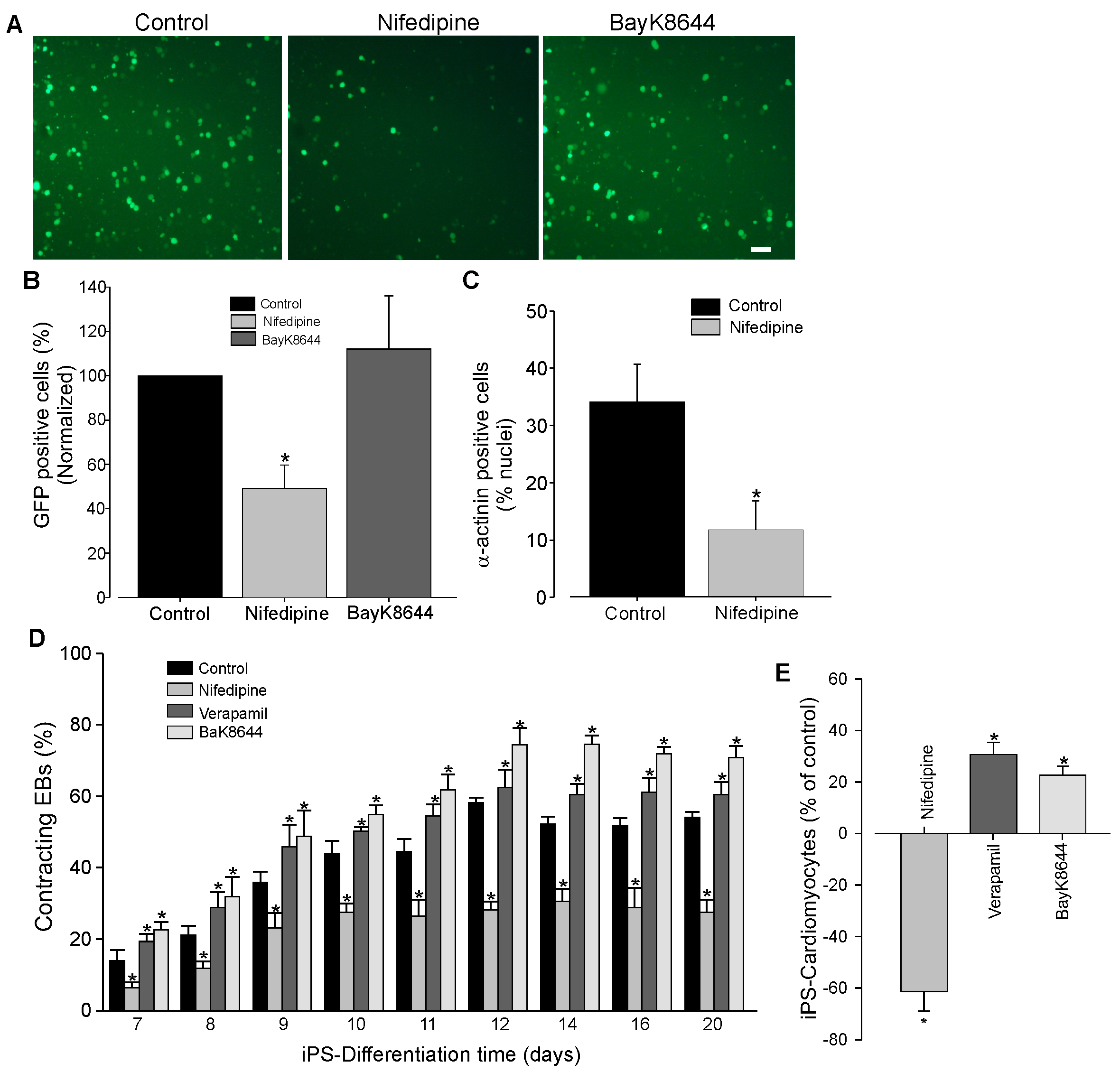

Supplement: Figure S1 — (A) Representative eGFP+ CMs obtained from dissociated EBs cultured under control conditions or in the presence of nifedipine or BayK8644. (B) eGFP-positive cells (CMs) at day 12 of differentiation generated under nifedipine- and BayK8644-treatment. CMs were manually counted and are expressed as a percentage of total CMs generated under control condition (C) The yield of CMs generated under control and nifedipine-treated conditions. Beating EBs were dissected, enzymatically dissociated and cultured on fibronectin- or gelatine-coated plates. Thereafter, cells were staining with nuclei dye Hoechst 33342 and CMs were investigated for α-actinin by means of immunocytochemistry. Alpha-actinin positive cells are represented as a percentage of total Hoechst 33432-labelled (positive nuclei) cells. (D)Time course of the incidence of ES and iPS cell-derived contracting EBs generated in the absence (control) or presence of nifedipine, verapamil or BayK8644. The mean±SEM of the percentage of EBs with contracting areas during differentiation is depicted. (E) Percentage of CMs (expressed as total of control) obtained by FACS analysis at day 12 of differentiation. Results are reported as the means±SEM (n = 3). * denote significant differences to control. Scale bars: 20 µm. (TIF) [file pone.0053407.s001.tif]

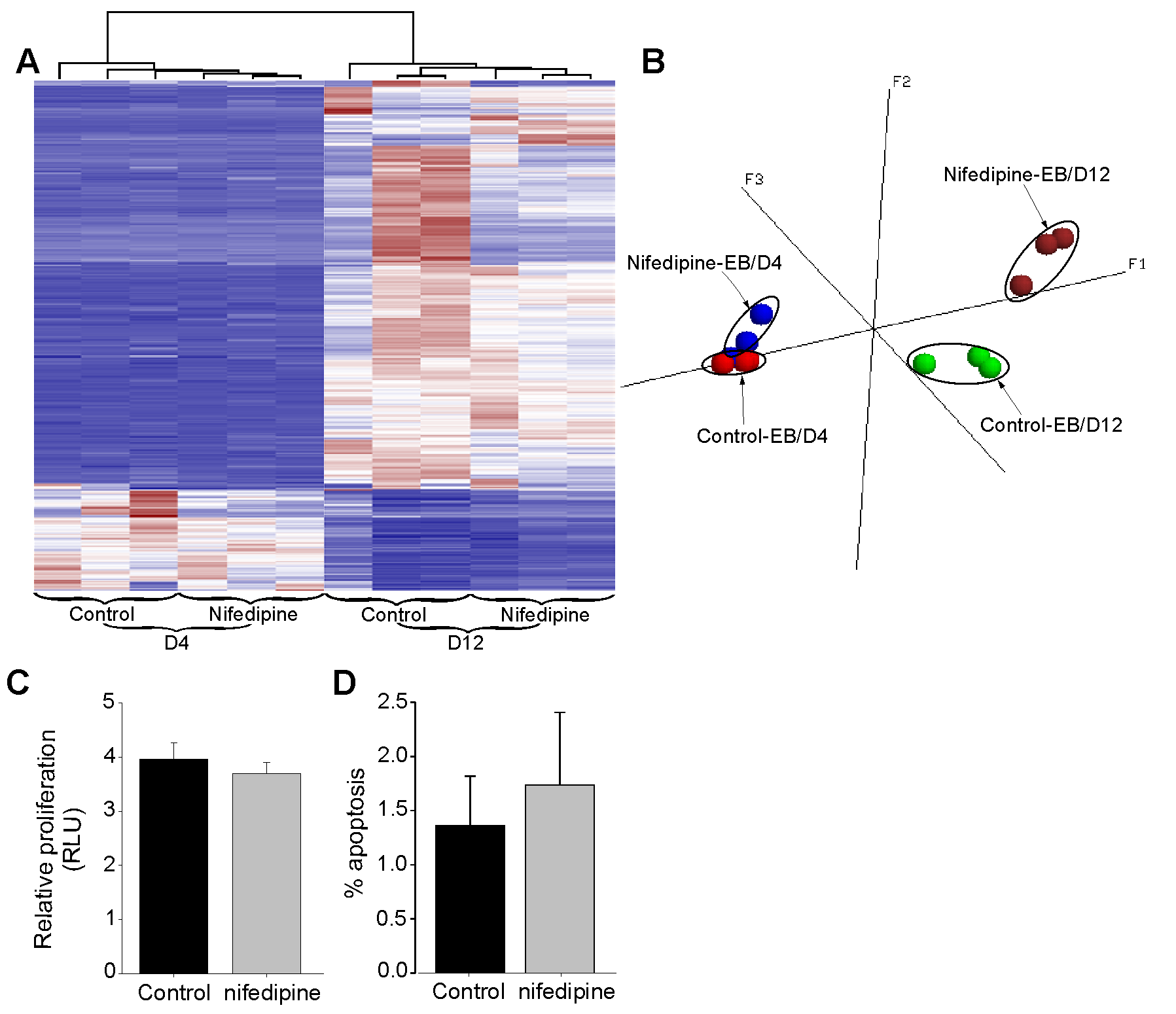

Supplement: Figure S2 — (A) Hierarchical clustering and (B) Principal component analysis of variable genes expression during induction of CMs under control and nifedipine-treated conditions at day 4 and 12 of differentiation. (C–D) Proliferation and viability assay of untreated and nifedipine-treated ES cells. Nifedipine did not alter cell proliferation (C) or viability (D) measured after 4 and 12 days of differentiation. A total of 3 independent experiments were studied for each time point (Scale bar, D: 50 µm and C: 200 µm). (TIF) [file pone.0053407.s002.tif]
