# Supplementary material for: The L-type Ca2+ Channels Blocker Nifedipine Represses Mesodermal Fate Determination in Murine Embryonic Stem Cells
Source: PLoS One. 2013 Jan 8;8(1):e53407. doi: 10.1371/journal.pone.0053407 (PMC3539992; doi:10.1371/journal.pone.0053407)
Supplement: Methods S1 — (DOC) [file pone.0053407.s006.doc]

**Nguemo et al**

**Online Supplemental Materials and Methods**

**Supplemental Methods**

**CMs Differentiation of ES and reprogrammed iPS cells**

Cardiac differentiation of ES and iPS cells was performed using the mass culture protocol as previously described [1], [3], [5]. Briefly, embryoid bodies (EBs) were first formed in non-adherent plates by culturing 1 x 10^6^ ES cells in 12 ml Iscove's Modified Dulbecco's Medium (IMDM) supplemented with 20% FCS (Invitrogen) in the presence or absence of nifedipine (10 µM), verapamil (10 µM) or BayK8644 (10 µM). The suspension culture dishes were placed on a horizontal shaker for 2 days to allow formation of EBs, were afterwards diluted to a density of about 400 EBs/12 ml and kept in differentiation medium. Nifedipine, verapamil and BayK8644 were prepared in 0.1% DMSO, stored in at -20°C and light protected also after addition to the cell cultures. Control cells were cultured in medium containing the same DMSO concentration as present in compound-treated cultures. Media was exchanged every second day. EGFP-positive areas in EBs were initially detected after 7–9 days of culture. To assess the efficacy of CMs differentiation, EBs were plated at day 7-9 on 0.1% gelatine-coated 12 well-plates with 20 EBs per well and monitored via fluorescence microscopy at day 9-12 for the presence of eGFP expression and calculation of spontaneous beating frequencies. For calculation of the beating frequency 8-10 day old EBs were plated on 12-microwell plates pretreated with 0.1% gelatin. For single CM experiments, contracting areas (CAs) were microdissected from EBs generated under control conditions and after nifedipine treatment, respectively, and were subsequently isolated by enzymatic dispersion using collagenase B (Roche, Mannheim, Germany) as described [1], [4].

**Single cell preparation**. Single cells were dissociated from undifferentiated cells (day 0) or whole EBs at day 4 to 8 of differentiation. Cardiomyocytes were dissociated from contracting areas (CAs) at 12 days of differentiation. The solution used for single cell dissociation was as follows (in mmol/L): 120 NaCl, 5.4 KCl, 5 MgSO_4_, 0.03 CaCl_2_, 5 Na pyruvate, 20 glucose, 20 taurine, 10 Hepes, and 1 mg/ml collagenase B, pH 6.9 (NaOH). The isolated cells were plated on sterile 0.1% gelatin-coated glass coverslips and cultured in Dulbecco´s Modified Eagle’s Medium (DMEM, Gibco) containing 15% fetal bovine serum. Isolated cells were used for immunocytochemical characterizations, Ca^2+^ imaging and electrophysiological experiments after 2-3 days of plating.

**RT-PCR and quantitative RT-PCR analysis**. We performed PCRs to study the specific gene expression from all 3 embryonic germ layers. For RT-PCR, total RNA was extracted from undifferentiated cells (day 0), EBs and CAs at day 12 of differentiation using the RNeasy mini kit (Qiagen GmbH, Germany) following the supplier's recommended protocol [3]. Briefly, cDNA was diluted 1:4 with sterile tri-distilled water and 5 µl were amplified using JumpStartTM RedTaq ReadyMixTM PCR Reaction Mix (Sigma). Negative controls were generated in RT reactions without RTase. Reactions were stopped at the exponential phase of amplification and products were analyzed by agarose gel electrophoresis.

For quantitative RT-PCR, total RNA was isolated using TRIzol Reagent (Invitrogen, Carlsbad, CA) from undifferentiated cells (day 0), EBs at day 2-5, 8 of differentiation and from 20-30 microdissected CAs (day 12). DNase I-treated total RNA (500 ng) was reverse-transcribed using Superscript II RTase (Invitrogen) and random hexamers. cDNA probes were diluted 1:10 and 2 µl was amplified using SYBR Green PCR Master Mix (Qiagen, Hilden, Germany) following the manufacturer's instructions. Real-time PCRs were performed in a 7500 Fast System Real Time Cycler (Applied Biosystems, Foster City, CA, USA) and analyzed with the SDSShell 1.4 software (Applied Biosystems). Relative expression was calculated according to the ΔΔCT method for quantification with the expression of GAPDH as housekeeping reference. Primers used and applied experimental RT-PCR conditions are depicted in detail in Supplemental Table 2. All experiments were repeated at least 3 times.

**Immunofluorescence**. Undifferentiated cells (day 0), whole EBs (day 12), Contracting areas (CAs) or dissociated single CMs were fixated with 4% paraformaldehyde, permeabilized with solution containing 0.25% Triton X-100 (Sigma-Aldrich) and 0.5 M ammonium chloride in 0.25 M TBS (pH 7.4) for 10 minutes and blocked with 0.8% bovine serum albumin (BSA) for 1 hour. For Ca_V_1.2 immunostaining, cells were fixated in methanol at −20°C for 10 hours, permeabilized with 0.5% Triton X-100 and blocked with 5% BSA for 1 h. Subsequently, samples were incubated overnight at 4°C with primary antibodies targeting the following: sarcomeric α-actinin (1:500) (Sigma-Aldrich) or Ca_V_1.2 (1:50) rabbit polyclonal anti-Ca_V_1.2 subunit (Alomone Labs, Israel). The preparations were incubated with the secondary antibodies anti-mouse-IgG1 (AlexaFluor555, 1:1000) or anti-rabbit-IgG1 (AlexaFluor647). Nuclei were counterstained with Hoechst 33342 (1:500, Sigma-Aldrich). After washing, samples were embedded in ProLong Gold Antifade Reagent and evaluated using a Zeiss Axiovert 200 epifluorescence microscope equipped with an ApoTome unit. For analysis the Zeiss AxioVision 4.5 software package (Zeiss, Göttingen, Germany) was used.

**Cell proliferation and viability assays.** Beating EBs from untreated and nifedipine-treated cultures at day 12 of differentiation were dissociated into single cell and used for the viability and proliferation assays. Cell proliferation was quantified using the CellTiter-Glo Luminescent Cell Viability Assay (Promega, Madison, USA) according to the manufacturer’s protocol. For viability assays, after dissociation, cells resuspended in 100 µl Annexin V binding buffer. Afterwards 5 µl of Annexin V-PE and 5 µl of 7-amino-actynomycin D (7-AAD) from the Annexin V-PE Apoptosis Detection Kit I (BD Biosciences) were added as also described.[6] After 15 min of incubation, 400 µl of Annexin V binding buffer was added before flow cytometry analysis. Fluorescence was detected by the FL3 channel. Data were analyzed by FACS using FACScan (BD Biosciences, Heidelberg, Germany). All experiments were performed in triplicate. Values were graphed as means ± standard deviations.

**Electrophysiology**. ES cells, EBs or CAs from both conditions were dissociated into single cells by using collagenase B or trypsin/EGTA, plated on gelatin-coated glass cover slips, and cultured for 24-48 hours before measurements. Patch-clamp pipettes, prepared from glass capillary tubes (Harvard Apparatus Ltd, Kent, UK) with a two-stage horizontal puller (DMZ Universal Puller, Munich, Germany), had a resistance of 2–3 MΩ when filled with intracellular solution. The glass coverslips containing the cells were placed into a temperature-controlled (37°C) recording chamber and perfused continuously with extracellular solution. Cell membrane capacitance was determined on-line using the Pulse software (Heka Electronic, Lambrecht, Germany). Action potentials (APs) of spontaneously beating CMs were recorded by the whole-cell current-clamp technique. The response of CMs to pharmacological intervention was assessed by administering isoproterenol (Iso) as ß-adrenoreceptor agonist, and carbachol (CCh) as the synthetic analogue of acetylcholine, a muscarinic receptor agonist. The voltage-clamp mode was used for recordings of currents. The extracellular recording solution contained (in mM): 140 NaCl, 5.4 KCl, 1.8 CaCl2, 1 MgCl2, 10 glucose, 10 HEPES, pH adjusted to 7.40 at 37°C with NaOH. The intracellular solution contained (in mM): 50 KCl, 80 K-aspartate, 1 MgCl2, 3 MgATP, 10 EGTA, and 10 HEPES, pH adjusted to 7.40 with KOH. APs were analyzed off-line with custom-made AP analysis software (provided by Prof. Philipp Sasse, University of Bonn, Germany).

In the voltage-clamp mode, we recorded voltage-gated Na^+^, L-type Ca^2+^, and depolarization-activated outward K^+^ channel currents. Current densities (pA) were determined to normalize the maximal current amplitude to cell size (pF).

For Na^+^ currents, the extracellular solution contained (in mM): 120 NaCl, 5 KCl, 3.6 CaCl_2_, 1 MgCl_2_, 20 TEA-Cl) and 10 HEPES, pH adjusted to 7.40 at 37°C with TEA-OH and the intracellular solution contained (in mM), 120 CsCl, 3 MgCl_2_, 5 MgATP, 10 EGTA, and 5 HEPES, pH adjusted to 7.40 with CsOH. Na^+^ currents were recorded at 5 mV voltage steps from -90 mV holding potential (HP) to -60/-55 mV (100 ms depolarization). Peak Na^+^ current was measured at -20 mV/ -15 mV and normalized to cell capacitance for current densities.

For recording K^+^ currents the extracellular solution contained (in mM): 135 N-methyl-D-glucamine (NMG) chloride, 5 KCl, 3.6 CaCl_2_, 1 MgCl_2_, 3 NiCl_2_, and 10 HEPES, pH adjusted to 7.40 at 37°C with NMG. The intracellular solution contained (in mM), 50 KCl, 80 K-aspartate, 1 MgCl_2_, 3 MgATP, 10 EGTA, and 10 HEPES, pH adjusted to 7.40 with NMG,. Depolarization-activated outward K^+^ currents were elicited by a family of 500 ms depolarizations from a –80-mV holding potential to voltages ranging from –60 to +60 mV (10 mV steps). We analyzed peak current values *I*_peak_ and sustained currents *I*_sus_ (remaining currents at the end of depolarization) as previously described [2].

For *I*_CaL_ ,the extracellular recording solution contained (in mM) 120 mM NaCl, 5 mM KCl, 3.6 mM CaCl_2_, 20 mM tetraethylammonium-Cl, 1 mM MgCl_2_, 10 mM HEPES, pH adjusted to 7.4 with TEA-OH. The internal solution contained (in mM) 120 CsCl, 3 MgCl_2_, 5 MgATP, 10 EGTA, 5 HEPES, pH adjusted to 7.4 with CsOH. Current was measured with a double-pulse protocol. First, a 100 ms depolarization from a holding potential of –90 mV to –40 mV was applied to inactivate Na^+^ and T-type Ca^2+^ channels, and then L-type Ca^2+^ channels were elicited by a family of 70 ms depolarization to voltages ranging from –60 to +50 mV (10 mV steps). The steady-state activation curve of Ca^2+^ current was constructed by normalizing the current density measured between −60 mV and 0 mV to that at 0 or 10 mV (peak of activation). Steady state inactivation was determined by stepping to various pre-pulse voltages (-60 to +50 mV, 10 mV increments) for 5 s prior to depolarization to a fixed test potential of +10 mV for 100 ms to evoke channel opening.

There was a 5 ms interpulse interval to allow resetting of the activation gate between the end of prepulse and the beginning of the test pulse. Steady-state inactivation curves were normalized to the current after the prepulse to −60 mV and averaged. Both, the steady state activation and inactivation curves, were fitted with a single Boltzmann equation (y = A2 + (A1-A2)/(1 + exp ((*V*_½_-x)/*k*)) that describes conductance (y) as a function of the potential (x), *V*_½_ as half-activating or half-inactivation potential, and *k* as the slope factor. The time constant (fast, τ_f_ and slow, τ_s_) phase of L-type Ca^2+^ current inactivation were estimated by fitting the current decay to a bi-exponential function using a simplex optimization algorithm.

A P/4 protocol was used in all experiments to subtract linear leak and capacitance. Series resistance (R_s_) and membrane capacitance (*C*_m_) were compensated to minimize the capacitive transient and routinely checked during the experiment. Only cells showing a stable C_m_ and R_s_ were included into analysis.

**Ca^2+^ imaging**

Isolated murine CMs were incubated for 12 min with 2 µM of the cell-permeable dye fura-2AM (Molecular Probes, Eugene, USA) at 37°C and then washed at least three times. De-esterification of the intracellular dye was allowed for 30 min at room temperature. Excitation light (340/380 nm) was applied using a monochromator at frequencies ranging from 2 to 5 Hz. The emitted fluorescence from the fura-2AM-loaded cells (>470 nm) was monitored using a charge-coupled device cooled camera (TILL Photonics, Planegg, Germany) and the emission data were analyzed using the Vision software package (TILL Photonics, Germany). The extracellular solution contained (in mMol/l) 140 NaCl, 5.4 KCl, 1.8 CaCl2, 1 MgCl2, 10 HEPES and 10 glucose (pH 7.4, adjusted with NaOH). Before being used for Ca^2+^ imaging experiments, CMs were repeatedly washed with standard extracellular solution to prevent any potentially remaining nifedipine effect. Results (changed in intracellular Ca^2+^-concentration) are displayed as 340/380 nm ratio calculated after background fluorescence subtraction. The Ca^2+^ content of SR stores was measured using rapid application of 10 mM caffeine as described [7]. Thus, the amplitude of the caffeine-induced Ca2+ transient in the cytoplasm was taken as the SR Ca2+ load. Fractional Ca2+ release from the SR was then calculated as the ratio of the amplitude of the stimulated cytoplasmic Ca2+ transient to SR Ca2+ load.

**Supplemental references**

1. Kolossov E, Fleischmann BK, Liu Q, Bloch W, Viatchenko-Karpinski S et al (1998) Functional characteristics of ES cell-derived cardiac precursor cells identified by tissue-specific expression of the green fluorescent protein. J Cell Biol 143:2045-2056

2. Lin S, Wang Z, Fedida D (2001) Influence of permeating ions on Kv1.5 channel block by nifedipine. Am J Physiol Heart Circ Physiol 280:H1160-1172

3. Mauritz C, Schwanke K, Reppel M, Neef S, Katsirntaki K et al (2008) Generation of functional murine cardiac myocytes from induced pluripotent stem cells. Circulation 118:507-517

4. Nguemo F, Sasse P, Fleischmann BK, Kamanyi A, Schunkert H et al (2008) Modulation of L-type Ca(2+) channel current density and inactivation by beta-adrenergic stimulation during murine cardiac embryogenesis. Basic Res Cardiol 104:295-306

5. Pfannkuche K, Liang H, Hannes T, Xi J, Fatima A et al (2009) Cardiac myocytes derived from murine reprogrammed fibroblasts: intact hormonal regulation, cardiac ion channel expression and development of contractility. Cell Physiol Biochem 24:73-86

6. Van Linthout S, Savvatis K, Miteva K, Peng J, Ringe J et al (2011) Mesenchymal stem cells improve murine acute coxsackievirus B3-induced myocarditis. Eur Heart J 32:2168-78

7. Raake PW, Zhang X, Vinge LE, Brinks H, Gao E, Jaleel N, Li Y, Tang M, Most P, Dorn GW, 2nd, Houser SR, Katus HA, Chen X, Koch WJ Cardiac G-protein-coupled receptor kinase 2 ablation induces a novel Ca2+ handling phenotype resistant to adverse alterations and remodeling after myocardial infarction. Circulation 125:2108-2118
